# Efficacy and safety of mosunetuzumab monotherapy for Japanese patients with relapsed/refractory follicular lymphoma: FLMOON-1

**DOI:** 10.1007/s10147-024-02662-5

**Published:** 2024-12-09

**Authors:** Hideki Goto, Takahiro Kumode, Yuko Mishima, Keisuke Kataoka, Yoshiaki Ogawa, Nobuhiro Kanemura, Kazuyuki Shimada, Toshiki Uchida, Yukano Kuroe, Atsuko Kawasaki, Jotaro Sato, Takanori Teshima

**Affiliations:** 1 Department of Hematology, Faculty of Medicine and Graduate School of Medicine, Hokkaido University, Hokkaido, Japan; 2 Division of Laboratory and Transfusion Medicine, Hokkaido University Hospital, Hokkaido, Japan; 3 Department of Hematology and Rheumatology, Faculty of Medicine, Kindai University, Osaka, Japan; 4 Department of Hematology Oncology, Japanese Foundation for Cancer Research, Cancer Institute Hospital, Tokyo, Japan; 5 Division of Hematology, Department of Medicine, Keio University School of Medicine, Tokyo, Japan; 6 Department of Hematology & Oncology, Tokai University School of Medicine, Kanagawa, Japan; 7 Department of Hematology and Infectious Disease, Gifu University Hospital, Gifu, Japan; 8 Department of Hematology and Oncology, Nagoya University Graduate School of Medicine, Aichi, Japan; 9 Department of Hematology and Oncology, Japanese Red Cross Aichi Medical Center Nagoya Daini Hospital, Aichi, Japan; 10 Chugai Pharmaceutical Co., Ltd, Tokyo, Japan

**Keywords:** Administration, Intravenous, Antibodies, Bispecific, Japan, Lymphoma, Follicular, Lymphoma, Non-Hodgkin

## Abstract

**Background:**

In a global phase I/II study (GO29781; NCT02500407), single-agent mosunetuzumab had a manageable safety profile and induced durable complete responses in patients with relapsed/refractory (R/R) B-cell non-Hodgkin lymphoma, including in patients with R/R follicular lymphoma (FL). In this analysis, the efficacy and safety of mosunetuzumab monotherapy were evaluated in an expansion cohort, FLMOON-1, in Japanese patients with R/R FL who had received  ≥ 2 prior lines of therapy in a phase I study (JO40295, jRCT2080223801).

**Methods:**

Mosunetuzumab was administered intravenously at the recommended phase II dose (with cycle 1 step-up dosing) for eight cycles or up to 17 cycles, or until disease progression or unacceptable toxicity. The pre-specified primary endpoint was Independent Review Facility (IRF)-assessed complete response rate (CRR; as best overall response). Secondary objectives included investigator (INV)-assessed CRR, INV- and IRF-assessed objective response rate (ORR), and safety.

**Results:**

At the data cutoff (October 13, 2023), 19 patients (median age 72 years) were evaluated. The IRF-assessed CRR and ORR were 68.4% and 78.9%, respectively; the INV-assessed CRR and ORR were 63.2% and 84.2%, respectively. Grade 3–4 adverse events (AEs) were observed in 89.5% of patients, with a low incidence of AEs leading to mosunetuzumab discontinuation (10.5%) and one fatal AE unrelated to mosunetuzumab. Cytokine release syndrome occurred in 47.4% of patients and were mostly Grade 1 in severity.

**Conclusion:**

These findings indicate mosunetuzumab has a consistent efficacy and manageable safety profile in Japanese patients with R/R FL compared with previously reported data from the global phase I/II study.

**Supplementary Information:**

The online version contains supplementary material available at 10.1007/s10147-024-02662-5.

## Introduction

Follicular lymphoma (FL), an indolent type of non-Hodgkin lymphoma, is considered incurable in most patients due to its typically relapsing–remitting pattern [1]. In Japan, FL is the second most common subtype of B-cell non-Hodgkin lymphoma (B-NHL), representing 13.5% of all cases [2]. The proportion of FL among B-cell neoplasms in Japan has gradually increased from 6% in 1996‒2000 to 22.4% in 2007‒2014, approaching the rates seen in Western countries (28–31%) [3].

FL is often characterized by relapsing disease, increasing refractoriness to anti-CD20 antibodies and chemotherapy, and decreasing progression-free survival (PFS) with each subsequent therapy [4]. There is no standard of care for relapsed/refractory (R/R) FL. Systemic treatments include a combination of antibody and chemotherapy, and high-dose chemotherapy followed by autologous stem-cell transplantation [5]. Further treatments, such as chimeric antigen receptor T-cell therapy (CAR-T), tazemetostat and rituximab–lenalidomide were recently approved in Japan; however, no reports confirm the relative superiority of these treatments. The introduction of CD19-targeted CAR-T showed encouraging efficacy in patients with R/R FL; however, its use may be limited by potentially severe toxicities and logistical challenges [6], limiting applicability for patients with rapidly progressive disease or high tumor burden. Effective new therapies with novel mechanisms of action are needed to improve outcomes for patients with R/R FL.

Mosunetuzumab is a CD20xCD3 T cell engaging bispecific antibody that redirects T cells to eliminate B cells, including those that cause malignant disease [7]. Mosunetuzumab is an off-the-shelf outpatient treatment approved by the United States Food and Drug Administration and the European Medicines Agency for patients with R/R FL who have received  ≥ 2 prior lines of therapy [8, 9].

In a global phase I/II study (GO29781; NCT02500407), single-agent intravenous (IV) mosunetuzumab administered with step-up dosing (SUD) in cycle 1 to mitigate the risk of cytokine release syndrome (CRS), had a manageable safety profile and induced durable complete responses in patients with R/R B-NHL. This included patients with R/R FL, who had received  ≥ 2 prior lines of therapy, and patients with a history of disease progression (PD) within 24 months from the start of initial therapy (POD24) [10–12]. Based on the observed efficacy and safety of mosunetuzumab following assessment of different doses and schedules, the recommended phase II dose (RP2D) with SUD was selected as 1 mg (Cycle [C]1 Day [D]1), 2 mg (C1D8), and 60 mg (C1D15 and C2D1) followed by 30 mg on D1 of subsequent cycles [10]. The efficacy and safety data from the global Phase I/II study have been compared with results from dose-escalation cohorts in Phase I of this study (JO40295, jRCT2080223801), assessing tolerability, safety, pharmacokinetics (PK), and efficacy of mosunetuzumab in 23 Japanese patients with R/R B-NHL [13]. The safety profile and antitumor activity of mosunetuzumab, as demonstrated in the global study, were similarly exhibited in Japanese patients with R/R B-NHL at the RP2D.

Given the encouraging data from the dose-escalation cohorts, Japanese patients with R/R FL who had received  ≥ 2 prior lines of therapy were enrolled to establish the efficacy and safety of mosunetuzumab monotherapy in this expansion cohort study.

## Materials and methods

### Study design

FLMOON-1 is a single-arm, dose-expansion cohort of the Japanese phase I bridging study (JO40295) of the global, open-label, phase I/II study (NCT02500407). Mosunetuzumab was administered with SUD in C1, as in Group B of the global phase I/II study [10, 11].

Adult patients (≥ 18 years) with histologically confirmed FL (Grade 1–3a) who had relapsed after, or failed to respond to, at least two prior lines of systemic therapy were eligible. FL diagnosis was confirmed locally prior to enrollment. Patients must have received prior treatment with an anti-CD20-directed therapy and an alkylating agent. Local testing of CD20 expression was mandatory prior to enrollment in the study. CD20 expression was confirmed retrospectively at the central laboratory. Full details of inclusion and exclusion criteria are included in the Supplementary Appendix (online only).

The protocol was approved by the relevant institutional review boards. The trial was conducted in accordance with the Declaration of Helsinki, the International Conference on Harmonization guidelines for Good Clinical Practice, and applicable laws and regulations. All patients provided written informed consent.

### Treatment

IV mosunetuzumab was administered in 21-day cycles at the RP2D with C1 SUD for the mitigation of CRS (C1D1: 1 mg; C1D8: 2 mg; C1D15 and C2D1: 60 mg; C3D1 + : 30 mg).

All patients received premedication with dexamethasone (20 mg IV) or methylprednisolone (80 mg IV) approximately 1 h before each mosunetuzumab dose until C3, where administration was optional. At the discretion of the investigator, patients received premedication with oral acetaminophen (500–1000 mg) and antihistamines (e.g., diphenhydramine hydrochloride 50–100 mg) 1 h before mosunetuzumab administration. Hospitalization after the mosunetuzumab injection was not mandatory.

Patients completed treatment if they achieved complete response (CR) after C8; those with a partial response (PR) or stable disease after C8 could continue mosunetuzumab monotherapy for up to 17 cycles, or until PD or unacceptable toxicity.

### Objectives and outcome assessments

The pre-specified primary endpoint was Independent Review Facility (IRF)-assessed CR (as best overall response) rate. Secondary objectives included investigator (INV)-assessed CRR, INV- and IRF-assessed objective response rate (ORR), duration of response (DoR), duration of complete response (DoCR), and PFS, overall survival (OS), PK and safety.

Computed tomography (CT) and ^18^F-fluorodeoxyglucose-positron emission tomography (PET) scans were performed at screening, 6 weeks (optional), 3 months (± 7 days), and 6 months (± 14 days) from the start of treatment, and every 3 months (± 14 days) thereafter. During post-treatment follow-up, CT with or without PET scans were done once every 3 months during the first 24 months, and once every 12 months thereafter until PD, start of new anti-cancer therapy, or study discontinuation. Tumor responses were assessed using the Revised Response Criteria for Malignant Lymphoma [14]. PD was assessed by PET or CT. Except for PD, responses with PET were prioritized; if no assessment with PET, assessment with CT was used.

Safety and tolerability were assessed by incidence, nature and severity of adverse events (AEs), changes in vital signs and laboratory parameters, and incidence of anti-drug antibodies. CRS events were reported based on published American Society for Transplantation and Cellular Therapy Criteria [15]; all other AEs were graded according to the National Cancer Institute Common Terminology Criteria for Adverse Events (NCI CTCAE) version 4.03 [16]. Serum mosunetuzumab PK parameters included area under the concentration–time curve (AUC) and maximum concentration (C_max_).

### Statistical analyses

For the primary analysis, IRF-assessed CRR was compared with a pre-specified historical control CRR. The control CRR was assumed to be 14% based on prospective FL data in the third line and beyond (3L +) from the phase II study of copanlisib as treatment for R/R indolent B-cell lymphoma [17]; if the lower limit of the 90% confidence interval (CI) for the CRR was higher than the predefined CRR of 14%, mosunetuzumab was considered to have demonstrated clinically significant efficacy. To detect an improvement in CRR, a sample size of 19 patients was required to achieve 94.4% overall power at a one-sided significance level of 5%. Primary efficacy analysis was performed after all on-treatment patients completed their 6-month tumor response assessment. The 90% and 95% CIs for CRR and ORR were calculated using the Clopper–Pearson method. DoR, DoCR, PFS, and OS were estimated using the Kaplan–Meier method; the 95% CI of the medians was calculated with the Brookmeyer–Crowley method. Six-month event-free rates were estimated, and the 95% CIs were calculated using the Greenwood formula.

## Results

### Patients

As of the clinical cutoff date (CCOD; October 13, 2023), 19 patients with ≥ 2 prior lines of therapy had been enrolled. Median age was 72 (range 58–80) years, 47.4% of patients were male and 84.2% had Stage III/IV disease (Table 1). The median number of prior lines of therapy was three (range 2–5). All patients (*N* = 19, 100%) were treated with anti-CD20 therapy and alkylator therapy, of whom 16 (84%) were treated with prior bendamustine. One (5.3%) patient was treated with immunomodulatory therapy, and one (5.3%) with CAR-T. Median mosunetuzumab dose intensity was 100% (range 63.6–100.8) and 11 (57.9%) patients received eight cycles of treatment. Median number of dosing cycles was eight (range 1–11).

**Table 1:** Patient demographics and baseline characteristics

*n* (%), unless stated	*N* = 19
Median age, years (range)	72.0 (58–80)
Male	9 (47.4)
Median body weight, kg (range)	59.8 (43.5–92.3)
ECOG PS	
0	17 (89.5)
1	2 (10.5)
Ann Arbor stage	
I/II	3 (15.8)
III/IV	16 (84.2)
FLIPI	
Low (0–1)	3 (15.8)
Intermediate (2)	4 (21.1)
High (3–5)	12 (63.2)
Bulky disease (> 6 cm)	
Yes	6 (31.6)
No	13 (68.4)
Median number of prior lines, *n* (range)	3 (2–5)
Prior systemic therapy	
Anti-CD20	19 (100)
Alkylator	19 (100)
IMiD	1 (5.3)
CAR-T	1 (5.3)
Refractory to prior anti-CD20 therapy and prior alkylator therapy	
Double refractory	8 (42.1)
Non-refractory	11 (57.9)
Refractory to prior anti-CD20 therapy	
Refractory	8 (42.1)
Non-refractory	11 (57.9)
Refractory to prior alkylator therapy	
Refractory	8 (42.1)
Non-refractory	11 (57.9)
Received prior CAR-T therapy	
Yes	1 (5.3)
No	18 (94.7)
Received prior rituximab and lenalidomide	
Yes	1 (5.3)
No	18 (94.7)
POD24	
Yes	5 (26.3)
No	11 (57.9)

*CAR-T* chimeric antigen receptor T cell, *ECOG PS* eastern cooperative oncology group performance status, *FLIPI* Follicular Lymphoma International Prognostic Index, *IMiD* immunomodulatory drug, *POD24* progression of disease within 24 months of first-line therapy

### Efficacy

**Fig. 1:**
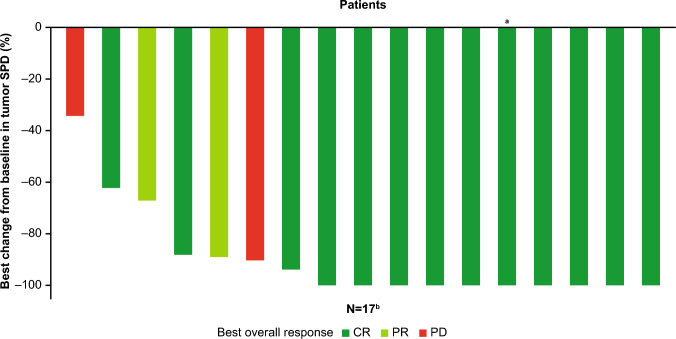
Best percentage change from baseline in tumor SPD. This waterfall plot shows the best overall change in the size of tumor target lesions according to the mosunetuzumab doses received with best overall response. Plots of the best percentage changes in the SPD of target lesions are shown. ^a^Patient with prior-CAR-T. ^b^Two patients out of 19 had no tumor assessment after baseline evaluation and were excluded from the analysis. *CR* complete response, *PD* progressive disease, *PR* partial response, *SPD* sum of product diameters

The IRF-assessed CRR was 68.4% (90% CI: 47.0–85.3; Table 2). The pre-specified primary endpoint was met, as the lower limit of the 90% CI exceeded the pre-specified threshold of 14%. IRF-assessed ORR was 78.9%; INV-assessed ORR and CRR were comparable with IRF-assessed rates (Table 2). Anti-tumor activity was observed in most patients; of the 17 patients who had at least one tumor evaluation after baseline, all experienced tumor reduction and 16 showed  ≥ 50% tumor reduction from baseline; the best percentage change from baseline in tumor sum of product diameters for each patient by response rate is presented in Fig. 1. IRF-assessed CRRs were generally consistent across patient subgroups (Supplementary Table 1). Among eight patients who were double refractory to anti-CD20 and alkylator therapy, six (75.0%) responded to treatment by IRF assessment. Of 11 patients who were non-double refractory, nine (81.8%) responded to treatment. Among 16 patients treated with prior bendamustine, 11 (68.8%) achieved CR and two (12.5%) achieved PR. One patient had received prior CAR-T and one prior rituximab and lenalidomide, with both patients having CR to mosunetuzumab treatment.

**Table 2:** Efficacy outcomes

Efficacy endpoints	*N* = 19^a^	
	IRF-assessed	INV-assessed
CRR, *n* (%) [90% CI, 95% CI]	13 (68.4) [47.0–85.3,43.4–87.4]	12 (63.2) [41.8–81.2,38.4–83.7]
ORR, *n* (%) [95% CI]	15 (78.9) [54.4–93.9]	16 (84.2) [60.4–96.6]
Median DoR, months [95% CI]	15.0 [3.7–15.0]	15.0 [3.4–15.0]
Median DoCR, months [95% CI]	13.6 [NE–NE]	13.6 [13.6–NE]
Median PFS, months [95% CI] 6-month PFS, % [95% CI]	16.4 [5.2–16.4] 76.0 [48.0–90.3]	16.4 [4.8–NE] 76.5 [48.8–90.5]
Median OS, months [95% CI] 6-month OS, % [95% CI]	NE [10.2–NE] 100 [100–100]	

*CI* confidence interval, *CRR* complete response rate, *DoR* duration of response, *DoCR* duration of complete response, *INV* investigator, *IRF* Independent Review Facility, *NE* not estimable, *ORR* objective response rate, *OS* overall survival, *PFS* progression-free survival

^a^Two patients without tumor assessment after baseline evaluation were evaluated as non-responders

**Fig. 2:**
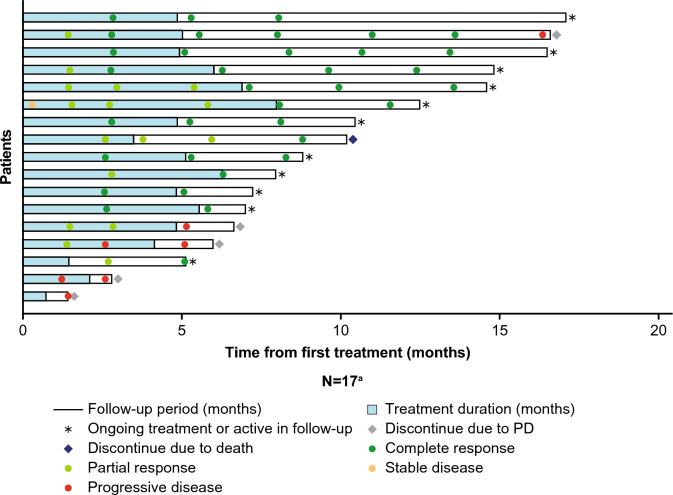
Time from first treatment with mosunetuzumab. ^a^Two patients out of 19 had no tumor assessment after baseline evaluation and were excluded from the analysis. *PD* progressive disease

Among 14 patients with a response, all responded by the first tumor assessment (~ 3 months after treatment initiation or earlier). Seven patients improved from PR to CR; once CR was achieved, it was generally maintained. As of the CCOD, 11 patients maintained CR after treatment ended. Duration and durability of treatment for each patient are presented in Fig. 2. After a median follow-up of 8.0 months, the median IRF-assessed DoR and DoCR were 15.0 months (95% CI: 3.7–15.0) and 13.6 months (95% CI: not estimable [NE]–NE), respectively. At 6 months, the event-free rate was 84.0% (95% CI: 48.7–95.9) among responders (Supplementary Fig. 1a), and 90.9% (95% CI: 50.8–98.7) among complete responders (Supplementary Fig. 1b). Median IRF- and INV-assessed PFS were 16.4 months (95% CI: 5.2–16.4) and 16.4 months (4.8–NE), respectively (Supplementary Fig. 1c; Table 2). OS data were immature (Table 2).

### Safety

Overall, 18 (94.7%) patients experienced any-grade AEs. Grade 3–4 AEs were observed in 89.5% of patients. The most common Grade 3–4 AEs were lymphocyte count decreased (68.4%), neutrophil count decreased (26.3%) and white blood cell count decreased (15.8%; Table 3). Eight serious AEs (SAEs) were reported by seven patients (36.8%); of these, six SAEs were considered related to mosunetuzumab (Table 3). There was one Grade 5 (fatal) AE (mosunetuzumab-unrelated small cell lung cancer [SCLC]; Table 3). AEs leading to mosunetuzumab discontinuation were uncommon (two patients [10.5%], one with SCLC [Grade 5] and one with immune effector cell-associated neurotoxicity syndrome [ICANS] [Grade 3], respectively; Table 3).

**Table 3:** Overall safety summary

*n* (%), unless stated	*N* = 19
All-grade AEs	18 (94.7)
Mosunetuzumab related^a^	18 (94.7)
Grade 3–4 AE	17 (89.5)
Mosunetuzumab related^a^	17 (89.5)
Lymphocyte count decreased^b^	13 (68.4)
Neutrophil count decreased^b^	5 (26.3)
White blood cell count decreased^b^	3 (15.8)
Infusion-related reaction^b^	2 (10.5)
Serious AE	7 (36.8)
Mosunetuzumab related^a^	5 (26.3)
Grade 5 (fatal) AE	1 (5.3)^c^
Mosunetuzumab related^a^	0
AE leading to discontinuation of treatment	2 (10.5)^d^
Mosunetuzumab related^a^	1 (5.3)^d^

*AE* adverse event, *ICANS* immune effector cell-associated neurotoxicity syndrome

^a^AE considered related to treatment by the investigator

^b^Mosunetuzumab-related Grade 3–4 AEs occurring in 10% or more of patients are shown

^c^Mosunetuzumab-unrelated small cell lung cancer

^d^Mosunetuzumab-related neurotoxicity/ICANS (one patient); mosunetuzumab-unrelated small cell lung cancer (one patient)

CRS events occurred in 47.4% of patients and were mostly Grade 1 in severity, except for one patient with a Grade 2 and one with a Grade 3 CRS event. CRS and infusion-related reaction was reported separately by medical judgment. Most CRS events occurred in C1, with only two events occurring in C2; the median time of onset of a CRS event was 13.5 h and the median duration was 2.0 days following the first dose (Supplementary Fig. 2). CRS was commonly observed on C1D1 (15.8%) and C1D15 (36.8%) when the first dose and loading dose (60 mg) of mosunetuzumab were administered, respectively. Among the nine patients who developed CRS, the patient with a Grade 3 event was 64 years old, had received four prior lines of therapy, and had bone marrow invasion, bulky disease, lactate dehydrogenase increased, ascites and pleural effusion as concomitant disease. Grade 3 CRS occurred after the C1D1 dose, and signs and symptoms of CRS were hypoxia and liver enzyme elevations, and the patient received high-flow oxygen, steroids, and tocilizumab. The patient with a Grade 2 event received fluids, steroids, and tocilizumab. The remaining seven patients (Grade 1 CRS) did not receive fluids, tocilizumab or steroids. No patient had to be admitted to the intensive care unit. All CRS events were resolved. There was no difference in the response to treatment for patients who did or did not experience a CRS event (Supplementary Table 2).

One patient experienced a Grade 3 AE reported as ICANS (decreased level of consciousness, incomprehensible speech). The initial symptoms of decreased level of consciousness occurred on D4 and were ameliorated the same day with initiation of dexamethasone. On D5, the patient experienced a seizure that subsided immediately following diazepam administration, as well as new symptoms of cramps in left arm and left side of face, tremors, and hiccups. After initiation of an anticonvulsant and methylprednisolone, these symptoms improved the following day. After a corticosteroid taper, seizures did not recur. ICANS was judged to have been resolved on D15.

Neutropenia was reported as neutrophil count decreased in 26.3% of patients; all events were resolved. No febrile neutropenia occurred. SAEs of infection occurred in one patient (COVID-19 pneumonia [Grade 2]; recovering). No tumor flare or tumor lysis syndrome events were observed.

### Pharmacokinetics

The mean (coefficient of variation [CV] %) AUC from D1–42 was 345 (26.5%) day* μg/mL (Supplementary Table 3). The mean (CV%) C_max_ from D1–42 was 26.2 μg/mL (27.8%). Steady state was reached at C3.

## Discussion

This dose-expansion cohort evaluated safety and efficacy of mosunetuzumab in Japanese patients with R/R FL and  ≥ 2 prior lines of therapy. Fixed-duration mosunetuzumab induced a high ORR and CRR with a manageable safety profile. Safety and efficacy data from this analysis were consistent with that previously reported in the global phase I/II GO29781 study [10, 11].

The follow-up period of this analysis was shorter than the global study (8.0 months vs 18.3 months); however, the proportion of patients who completed eight cycles of treatment after achieving CR at C8 was similar [11]. In this analysis, mosunetuzumab demonstrated a high CRR (68.4%) and ORR (78.9%), including a high CRR in patients with POD24 (80.0%), and response to treatment was observed regardless of prior therapy and refractoriness. In one patient who had received prior CAR-T, CR following mosunetuzumab treatment was observed, with no CRS. While CRRs were similar across subgroups, the small number of patients in each subgroup limits the reliability of these comparisons. Eleven (57.9%) patients did not experience POD24. Both DoR and DoCR at 6 months were consistent with the results of the global study [10, 11]; however, due to limited follow-up, it is necessary to interpret the median values with caution. Due to the limited number of patients, no firm conclusions for PFS or OS can be made.

Overall, mosunetuzumab monotherapy demonstrated a manageable safety profile. The AE profile and severity of AEs were consistent with the known safety profile of mosunetuzumab [8–11]. Of note, a higher incidence of Grade 3–4 AEs was reported in this analysis versus the global phase I/II GO29781 study [10, 11]. This could be attributed to the difference in incidence of lymphocyte count decreased/lymphopenia, which is associated with the mode of action of mosunetuzumab, and unlike this analysis was not frequently reported in the global study.

The incidence of AEs leading to mosunetuzumab discontinuation was low. (*n* = 2; 10.5%), and only one Grade 5 (fatal) AE, which was considered unrelated to mosunetuzumab, was reported. The rate of neutropenia/neutrophil count decrease was comparable between this and the global study (26.3% and 28.9%, respectively) [11]. The current trial was held during the COVID pandemic, and one serious event of COVID-19 pneumonia was reported; no febrile neutropenia events and no Grade 5 (fatal) AEs due to infection were reported. Neurological AEs consistent with ICANS were rare in both studies, one patient in the current study and 1% of patients (Grade 1: 0.5%; Grade 2: 0.5%) who received mosunetuzumab at the recommended dose in the global trial [11]. Overall, no new safety findings were observed, which was expected given the tolerable mosunetuzumab dosing regimen established in the global phase I/II GO29781 study [10, 11].

The incidence of CRS was similar between this and the global phase I/II GO29781 study, with any-grade CRS events observed in 47.4% and 44.4% of patients, respectively [11]. Most CRS events in this study were Grade 1 (36.8%) and were lower grade versus the global study, with steroids as the premedication for CRS at C1 and C2 in both studies [11]. Consistent with the global study, responses to treatment were observed in patients regardless of whether they experienced a CRS event [11]. This suggests that the use of SUD decreased the risk of CRS, while CRS events do not influence the clinical efficacy of mosunetuzumab. The use of tocilizumab and steroids to mitigate CRS was comparable between the two studies.

The blood concentration of mosunetuzumab increased in a dose-dependent manner in this study [13]. While the half-life of mosunetuzumab was not evaluated in this dose-expansion cohort due to limited blood sampling points, the apparent half-life of the first dosing for Japanese patients has been previously reported [13]. The mean C_max_ (CV%) and mean AUC (CV%) at the RP2D were 26.2 μg/mL (27.8%) and 345 (26.5%) day*μg/mL, respectively, compared with 17.9 μg/mL (49.6%) and 246 (46.9%) day*μg/mL, respectively, at the same RP2D in Group B in the global phase I/II study [18]. Although a trend toward higher exposure was observed in this analysis compared with the global phase I/II GO29781 study, the differences observed are likely due to the difference in body weight between the two studies. Pharmacokinetic data in this dose-expansion cohort are consistent with those observed in the Japanese Phase I study [13].

In the current study, the correlation between response and peripheral T cells was not investigated; however, no association between baseline B cell, T cell, or NK-cell counts, and response was observed in the global phase I/II GO29781 study [11].

Limitations of this analysis include a lack of control group and the small number of Japanese patients enrolled (*N* = 19). The duration of follow-up in this study was relatively short (8.0 months); however, results from the global phase I/II GO29781 study, with longer follow-up, demonstrate efficacy of mosunetuzumab treatment with a long DoR and manageable safety [11].

Given the lack of clear evidence for treating 3L + FL with various treatments, including CAR-T, in clinical practice, prolonged follow-up of mosunetuzumab may be considered for future studies to extend upon the efficacy findings of this analysis.

## Conclusion

Findings from FLMOON-1 demonstrate a high CRR with mosunetuzumab monotherapy, a fixed-duration and off-the-shelf treatment, in Japanese patients with R/R FL and  ≥ 2 prior lines of therapy. A trend toward higher treatment exposure than that in the global Phase I/II GO29781 study was observed. The consistent efficacy and manageable safety profile of mosunetuzumab in Japanese patients with 3L + FL, compared with the global phase I/II study, expand on and add weight to the previous findings.

## Supplementary Information

Below is the link to the electronic supplementary material.

Supplementary file1 (DOCX 671 KB)

## Data Availability

Qualified researchers may request access to individual patient-level data through the clinical study data request platform (www.clinicalstudydatarequest.com. For further details on Chugai’s Data Sharing Policy and how to request access to related clinical study documents, see www.chugai-pharm.co.jp/english/profile/rd/ctds_request.html.
