# Supplementary material for: Efficacy and safety of mosunetuzumab monotherapy for Japanese patients with relapsed/refractory follicular lymphoma: FLMOON-1
Source: Int J Clin Oncol. 2024 Dec 9;30(2):389–96. doi: 10.1007/s10147-024-02662-5 (PMC11785659; doi:10.1007/s10147-024-02662-5)
Supplement: Supplementary file 1 — Supplementary file1 (DOCX 671 KB) [file 10147_2024_2662_MOESM1_ESM.docx]

**Supplementary Appendix**

**Efficacy and safety of mosunetuzumab monotherapy for Japanese patients with relapsed/refractory follicular lymphoma: FLMOON-1**

**Authors:** Hideki Goto,^1,2^ Takahiro Kumode,^3^ Yuko Mishima,^4^ Keisuke Kataoka,^5^ Yoshiaki Ogawa,^6^ Nobuhiro Kanemura,^7^ Kazuyuki Shimada,^8^ Toshiki Uchida,^9^ Yukano Kuroe,^10^ Atsuko Kawasaki,^10^ Jotaro Sato,^10^ Takanori Teshima^1,2^

**Affiliations:** ^1^Department of Hematology, Faculty of Medicine and Graduate School of Medicine, Hokkaido University, Hokkaido, Japan; ^2^Division of Laboratory and Transfusion Medicine, Hokkaido University Hospital, Hokkaido, Japan; ^3^Department of Hematology and Rheumatology, Faculty of Medicine, Kindai University, Osaka, Japan; ^4^Department of Hematology Oncology, Japanese Foundation for Cancer Research, Cancer Institute Hospital, Tokyo, Japan; ^5^Division of Hematology, Department of Medicine, Keio University School of Medicine, Tokyo, Japan; ^6^Department of Hematology & Oncology, Tokai University School of Medicine, Kanagawa, Japan; ^7^Department of Hematology and Infectious Disease, Gifu University Hospital, Gifu, Japan; ^8^Department of Hematology and Oncology, Nagoya University Graduate School of Medicine, Aichi, Japan; ^9^Department of Hematology and Oncology, Japanese Red Cross Aichi Medical Center Nagoya Daini Hospital, Aichi, Japan; ^10^Chugai Pharmaceutical Co., Ltd, Tokyo, Japan

**Author for correspondence:**
Hideki Goto
Department of Hematology, Faculty of Medicine and Graduate School of Medicine, Hokkaido University, N-15, W-7, Kita-ku, Sapporo, Hokkaido 060-8638, Japan
Tel: +81-11-706-7214
E-mail: [hidekigt@med.hokudai.ac.jp](mailto:hidekigt@med.hokudai.ac.jp)

**Target journal:** International Journal of Clinical Oncology

**Inclusion** **and Exclusion criteria**

**Inclusion criteria**

- Provided written informed consent after having received a thorough explanation of this study
- Aged ≥18 years at the time of informed consent
- Eastern Cooperative Oncology Group performance status of 0 or 1
- Life expectancy of at least 12 weeks after the day of enrollment
- Histologically confirmed relapsed or refractory CD20-positive, Grade 1–3a follicular lymphoma
- Received at least two prior regimens of systemic lymphoma therapy including an anti-CD20 targeted therapy and an alkylating agent
- Able to provide tumor tissue samples meeting all of the criteria below:
  - Formalin-fixed, paraffin-embedded blocks of tumor tissue samples or three unstained slides
  - Sample taken after the last administration of the previous therapy (however, if this procedure could not be performed, substitution with a sample taken before the last administration of the previous therapy maybe allowed after consultation with the sponsor)
  - No evidence of phenotypic transformation or change in histological type since the time of tumor tissue sampling
- At least one measurable lesion (measurable in two perpendicular directions on CT or MRI imaging with longest diameter >1.5 cm in nodal disease or >1.0 cm in extranodal disease)
- Major organ functions meeting all of the criteria below. (Except for findings judged by the investigator to the concomitant disease or other conditions [eg, immune thrombocytopenia] and/or bone marrow involvement). If multiple test results are available during this period, those obtained closest to enrollment will be used
  - Neutrophil count: ≥1,000 /μL
  - Platelet count: ≥75,000 /μL
  - Hemoglobin: ≥9.0 g/dL
  - Aspartate transaminase and alanine transaminase: ≤3 times the upper limit of the site reference range
  - Total bilirubin: ≤1.5 times the upper limit of the site reference range; patients with a history of Gilbert’s syndrome who are allowed to enroll if elevated total bilirubin is accompanied by elevated indirect bilirubin.
  - Serum creatinine: ≤ the upper limit of the site reference range or creatinine clearance ≥60 mL/min. Directly measured or calculated using the Cockcroft-Gault formula:
    - Men: [(140 – age) × Body weight (kg)] / [72 × Serum creatinine (mg/dL)]
    - Women: [(140 – age) × Body weight (kg) × 0.85] / [72 × Serum creatinine (mg/dL)]
- By the scheduled Cycle 1 Day 1, at least the following period of time must have passed since the previous treatments or interventions indicated below:
  - Surgery: 4 weeks. This applies to major surgeries such as those requiring thoracotomy, laparotomy, or associated with laparoscopic organ resection; however, if biopsies (tumor biopsy and bone marrow biopsy) prescribed in the protocol are performed within this period, patients who are judged by the investigator to have recovered from the surgery and be at no risk of bleeding at the time of enrollment may be enrolled in the study
  - Chemotherapy: five half-lives of the drug or 4 weeks, whichever is shorter (including molecular-targeted drugs)
  - Antibody therapy: 4 weeks (including monoclonal antibodies, radioimmunoconjugates and antibody-drug conjugates). Excluding the following cancer immunotherapies:
    - Cancer immunotherapy: five half-lives of the drug or 12 weeks, whichever is shorter (immune checkpoint inhibitors [e.g., anti-PD-1/PD-L1 antibody drugs, anti-CTLA-4 antibody drugs, etc.] or co-stimulatory agonists [e.g., anti-CD137 antibody drugs, anti-CD27 antibody drugs, anti-GITR antibody drugs, anti-CD40 antibody drugs, etc.] and all other cancer immunotherapies [including immunomodulators such as IFN-α, IFN-γ, IL-2, BRM and cancer vaccine therapy, etc.])
  - Radiotherapy: 2 weeks
    - If there is only one measurable lesion and no clear progression of the irradiated lesion: 4 weeks
  - Autologous hematopoietic stem cell transplantation: 12 weeks
  - Platelet transfusion: 2 weeks
  - Red blood cell transfusion: 3 weeks
  - Immunosuppressive therapy: 2 weeks

including prednisolone, cyclophosphamide, azathioprine, methotrexate, thalidomide and anti-TNF-α agents; however, continued use of the following immunosuppressive therapies are allowed:

- - - Inhaled steroids
    - Prednisolone at ≤10 mg/day or equivalent corticosteroids
    - Systemic immunosuppressants for acute illness (nausea, B symptoms, etc.)
    - Mineralocorticoids for the management of orthostatic hypotension
    - Corticosteroids for the management of adrenal insufficiency
  - Live vaccine, attenuated vaccine, inactivated vaccine, toxoid: 4 weeks
  - Alemtuzumab, fludarabine, cladribine, pentostatin: 24 weeks; unless fludarabine was used as lymphodepleting therapy prior to chimeric antigen receptor T-cell (CAR T-cell) therapy
  - Other investigational products: five half-lives of the drug or 4 weeks, whichever is shorter
  - CAR T-cell therapy: 30 days

**Exclusion criteria**

Patients who met any of the following criteria were excluded from study entry:

- History of hypersensitivity to the excipients of mosunetuzumab, including histidine acetate, sucrose, polysorbate and L-methionine
- History of severe allergic or anaphylactic reactions to monoclonal antibody therapy (chimeric antibodies, humanized antibodies, human antibodies) or fusion proteins
- History or presence of central nervous system (CNS) lymphoma
- History of malignancy that could affect compliance with the protocol or interpretation of results. With the exception of the following malignancies that have been previously treated curatively:
  - A history of curatively treated basal or squamous cell carcinoma of the skin, carcinoma in situ of the cervix, or ductal carcinoma in situ of the breast with favorable prognosis
  - Stage I melanoma, low-grade early stage localized prostate cancer
  - Other previously treated malignancies that have been in remission without treatment for at least 2 years prior to first dose
    of mosunetuzumab
- Persistent treatment-emergent adverse reactions associated with prior therapy that are rated Grade ≥2 according to the National Cancer Institute Common Terminology Criteria for Adverse Events (NCI CTCAE) version 4.03; however, patients with alopecia and lymphocyte count decreased are allowed
- History of the following immune-mediated adverse events related to the past immunotherapy:
  - Events of Grade ≥3 according to NCI CTCAE v4.03 (excluding endocrine disorders which are controllable with hormone replacement therapy)
  - Any NCI CTCAE v4.03 Grade 1/2 events that did not resolve after discontinuation of immunotherapy
- Current active infectious diseases requiring systemic administration of antimicrobial, antifungal, or antiviral drugs, etc., or history of these diseases within 4 weeks prior to enrollment; however, patients with superficial fungal infection are allowed It is recommended that infection be confirmed through SARS-CoV-2 PCR testing or antigen testing.
- Current or past history of autoimmune disease; however, patients meeting any of the following conditions are allowed:
  - History of autoimmune disease or well-controlled autoimmune disease, with at least 12 months since the last immunosuppressive therapy, may be eligible if deemed safe by the investigator
  - History of hypothyroidism controlled with stable doses of thyroid replacement hormone
  - History of disease-related immune thrombocytopenic purpura or autoimmune hemolytic anemia
  - Skin rash ≤10% of body surface area (remains in skin lesions such as eczema, psoriasis, lichen simplex chronicus and leukoderma), controlled with low-potency topical corticosteroids, with no acute exacerbation within 12 months of enrollment, and not requiring psoralen, ultraviolet A radiation, methotrexate, retinoids, biologic agents, oral calcineurin inhibitors, or high-potency corticosteroids
- Current active tuberculosis
- Prior allogenic stem cell or organ transplantation
- Positive test results for human immunodeficiency virus antibodies
- Positive test results for HBs antigen, HBs antibodies, or HBc antibodies; however, patients in whom positivity for HBs antibodies is clearly attributable to vaccination and patients who are positive only for HBs antibodies and/or HBc antibodies, and negative HBV-DNA can be enrolled
- Positive test results for HCV antibodies; however, patients who are positive for HCV antibodies and negative for HCV-RNA can be enrolled
- Evident or suspected chronic active Epstein Barr Virus infection
- History of Progressive Multifocal Leukoencephalopathy
- History or current diagnosis of hemophagocytic lymphohistiocytosis or macrophage activation syndrome
- Presence of clinically significant and poorly controlled lung diseases that can affect the interpretation of the study results, such as current or history of lung disease requiring treatment, e.g., obstructive pulmonary disease or symptomatic bronchospasms
- Current cardiac diseases, arrhythmias, or unstable angina included in the New York Heart Association Class III or higher, or history of myocardial infarction within 6 months prior to enrollment
- Current or history of CNS diseases (stroke, epilepsy, CNS vasculitis, neurodegenerative disease, etc.)
  - Patients with a history of stroke who have not experienced a stroke or transient ischemic attack in the past 2 years, and with no residual neurological deficit in the opinion of the investigator, may be enrolled
  - Patients with a history of epilepsy who have not had seizures in the past 2 years while not being treated with antiepileptic drugs may be enrolled
- Pregnant or lactating women (except for lactating women who have consented to stop breast feeding over a period from the start of treatment with the investigational product to the time point of 90 days after the final administration of mosunetuzumab or tocilizumab, whichever is longer)
- Women with a positive pregnancy test result (women who are not post-menopausal [≥12 months of amenorrhea due to unidentified causes except for menopause] and have not undergone ovariectomy and/or hysterectomy will be regarded as women of child-bearing potential and must take a pregnancy test. Women who had experienced amenorrhea in the last 12 months before enrollment but could still be pregnant because the amenorrhea was chemically induced or due to other relevant reasons must also take a pregnancy test)
- Women of child-bearing potential who have no intention to be abstinence (avoiding heterosexual intercourse), have no intention to use an appropriate method of contraception with an annual failure rate of <1%, or have no intention to provide egg donation during the period of this study and for 90 days after the last dose of mosunetuzumab or tocilizumab, whichever is longer
- Men who have no intention to be abstinence (avoiding heterosexual intercourse), have no intention to use an appropriate method of contraception with an annual failure rate of <1%, or have no intention to provide sperm donation during the period of this study and for 60 days after the last dose of mosunetuzumab or for 90 days after the last dose of tocilizumab, whichever is longer
- History of previous treatment with a bispecific anti-CD20 antibody targeting both CD20 and CD3
- Other patients who are judged by the investigator to be ineligible for participation in this study

**Supplementary Table 1** Subgroup analysis of IRF-assessed CR rate

|  | **Number of patients by subgroup** | **Number of patients with CR** | **CR, % (95% CI)** |
| --- | --- | --- | --- |
| All | 19 | 13 | 68.4 (43.5–87.4) |
| Sex  Male  Female | 9  10 | 5  8 | 55.6 (21.2–86.3)  80.0 (44.4–97.5) |
| Age  <65  ≥65 | 5  14 | 4  9 | 80.0 (28.4–99.5)  64.3 (35.1–87.2) |
| ECOG PS  0  1 | 17  2 | 13  0 | 76.5 (50.1–93.2)  0 (0.0–84.2) |
| FLIPI  Low (0–1)  Intermediate (2)  High (3–5) | 3  4  12 | 3  4  6 | 100 (29.2–100)  100 (39.8–100)  50.0 (21.1–78.9) |
| Bulky disease (>6 cm)  Yes  No | 6  13 | 4  9 | 66.7 (22.3–95.7)  69.2 (38.6–90.9) |
| No. of prior lines of therapy  ≥3  2 | 12  7 | 8  5 | 66.7 (34.9–90.1)  71.4 (29.0–96.3) |
| Refractory to last line of therapy  Refractory  Non-refractory | 9  10 | 5  8 | 55.6 (21.2–86.3)  80.0 (44.4–97.5) |
| Refractory to prior anti-CD20 therapy  Refractory  Non-refractory | 8  11 | 6  7 | 75.0 (34.9–96.8)  63.6 (30.8–89.1) |
| Refractory to prior alkylator therapy  Refractory  Non-refractory | 8  11 | 6  7 | 75.0 (34.9–96.8)  63.6 (30.8–89.1) |
| Double-refractory to prior anti-CD20 therapy and alkylator therapy  Refractory  Non-refractory | 8  11 | 6  7 | 75.0 (34.9–96.8)  63.6 (30.8–89.1) |
| Received prior CAR-T therapy  Yes  No | 1  18 | 1  12 | 100 (2.5–100.0)  66.7 (41.0–86.7) |
| Received prior rituximab and lenalidomide  Yes  No | 1  18 | 1  12 | 100 (2.5–100.0)  66.7 (41.0–86.7) |
| POD24  Yes  No | 5  11 | 4  7 | 80.0 (28.4–99.5)  63.6 (30.8–89.1) |

*CAR-T* chimeric antigen receptor T-cell, *CI* confidence interval, *CR* complete response, *ECOG PS* Eastern Cooperative Oncology Group performance status, *FLIPI* Follicular Lymphoma International Prognostic Index, *IRF* Independent Review Facility, *POD24* progression of disease within 24 months

**Supplementary Table 2** IRF-assessed best response by number of patients with or without CRS events^a^

| Response, *n* | **Patients with CRS events**  ***n* = 9** | **Patients without CRS events**  ***n* = 10** |
| --- | --- | --- |
| Responder (CR + PR)  CR  PR | 7  5  2 | 8  8  0 |
| Non-responder  SD  PD  NE | 1  0  1  1 | 1  0  1  1 |

*CR* complete response, *CRS* cytokine release syndrome, *IRF* Independent Review Facility, *NE* not evaluable, *PD* progressive disease, *PR* partial response, *SD* stable disease

^a^Infusion-related reaction was reported when distinguished from CRS by medical judgement

**Supplementary Table 3** Pharmacokinetic parameters of mosunetuzumab in Japanese patients

|  | **T*_max_*** | **C*_max_***  **(µg/mL)** | **AUC*_0-42_***  **(day*µg/mL)** |
| --- | --- | --- | --- |
| N | 13 | 13 | 12 |
| Mean | 21.0 | 26.2 | 345 |
| SD (CV%) | 0.34 (1.6) | 7.28 (27.8) | 91.3 (26.5) |
| Median | 21.1 | 26.2 | 345 |
| Min–Max | 20.2–21.2 | 11.3–38.3 | 147–474 |

*AUC_0-42_*, area under Cycle 1 and Cycle 2 (42 days) serum concentration curve; *C_max_*, maximum serum concentration from 0 to 42 days; *CV*, coefficient of variation; *T_max_*, time from dosing to maximum concentration; *SD*, standard deviation


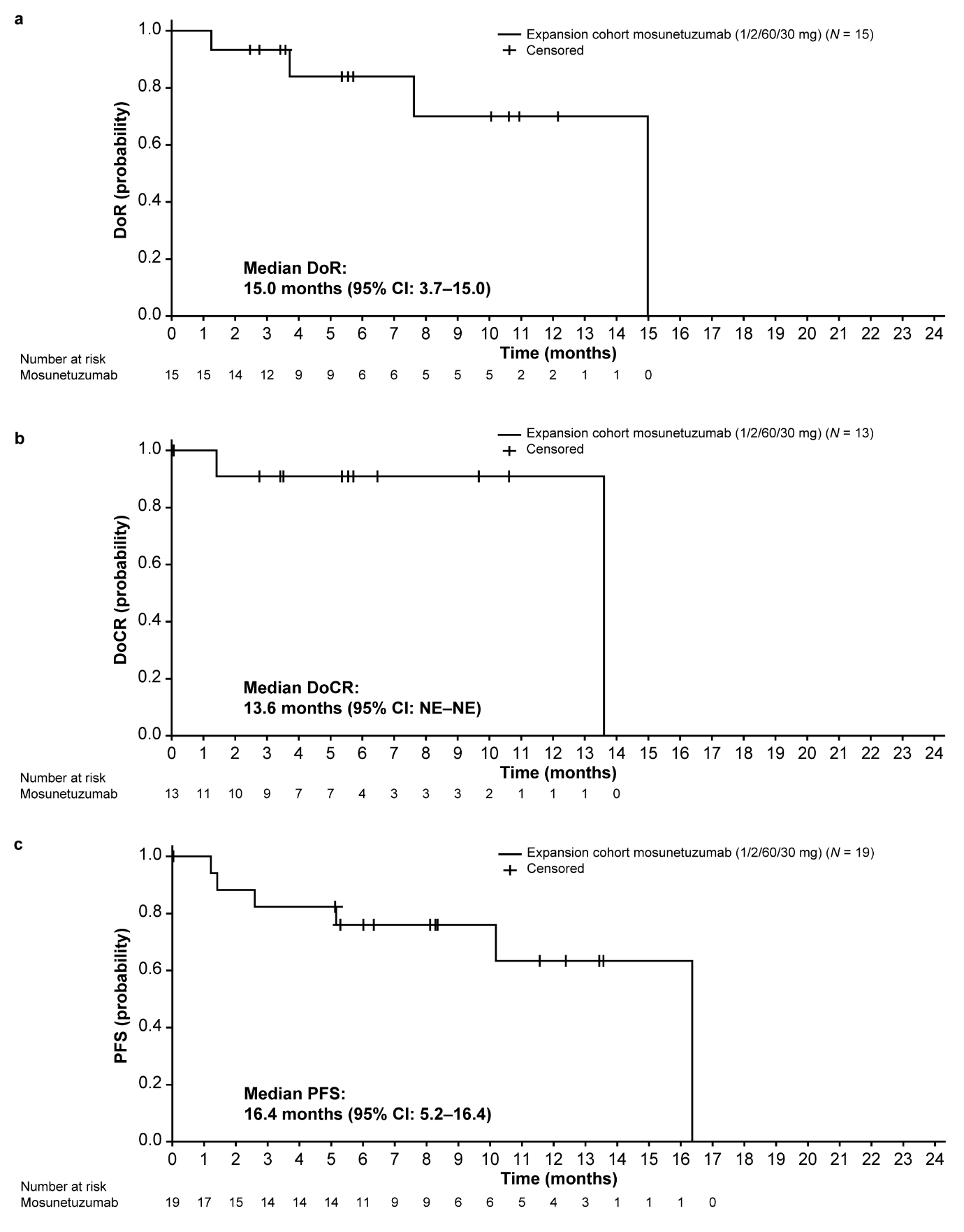


**Supplementary Fig. 1** Kaplan-Meier estimates of IRF-assessed (**a**) DoR, (**b**) DoCR, and (**c**) PFS. *CI* confidence interval, *DoR* duration of response, *DoCR* duration of complete response, *IRF* Independent Review Facility, *NE* not estimable, *PFS* progression-free survival


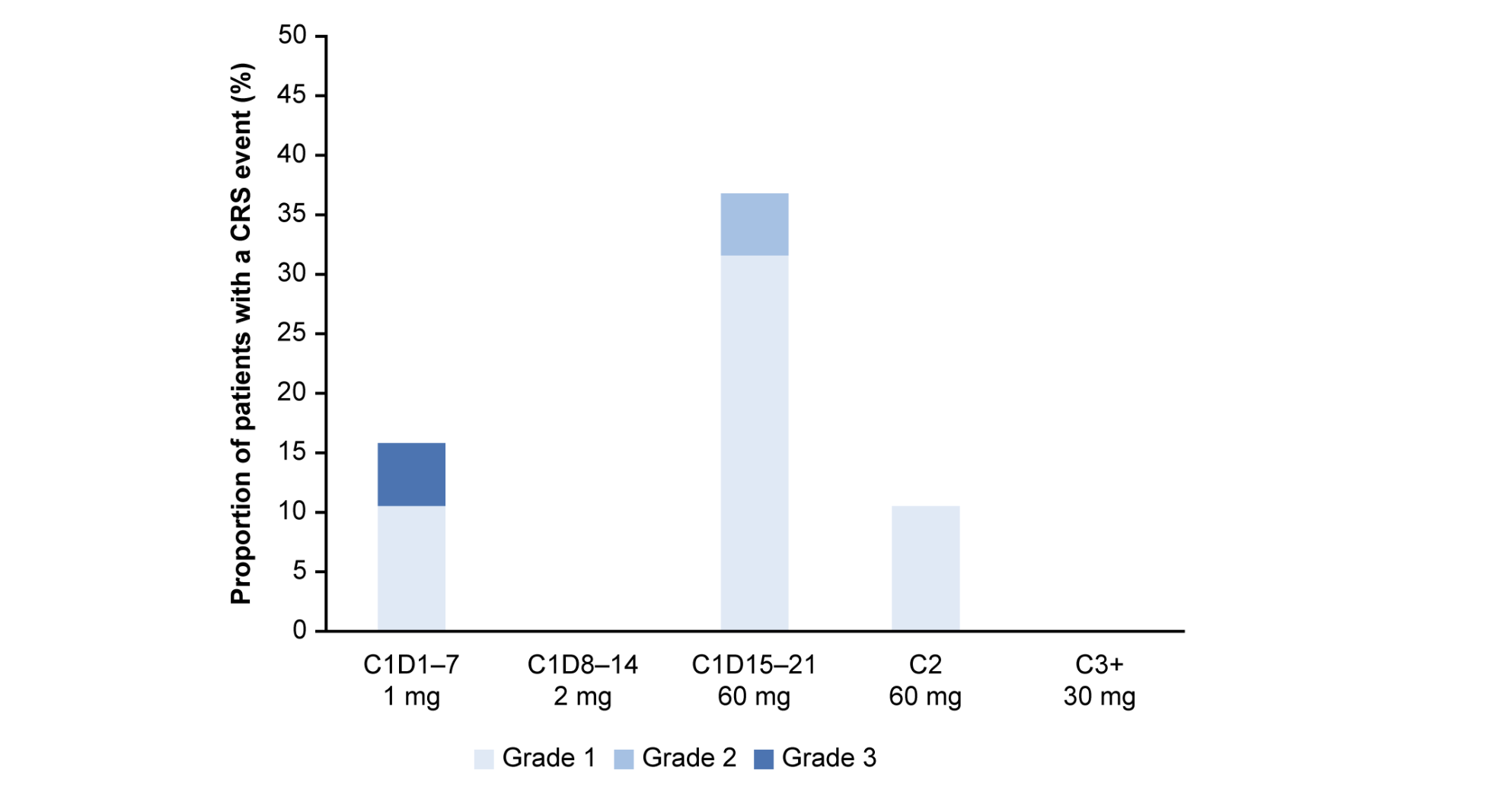


**Supplementary Fig. 2** CRS by cycle and grade. *C* cycle, *CRS* cytokine release syndrome, *D* day
